# Photogeneration and Visualization of a Surface‐Stabilized Dinitrene

**DOI:** 10.1002/anie.202502640

**Published:** 2025-03-06

**Authors:** Federico Frezza, Ana Sánchez‐Grande, Sofia Canola, Christophe Nacci, Jiří Klívar, Pingo Mutombo, Qifan Chen, José María Gómez‐Fernandez, Carlos Sánchez‐Sánchez, Jan Berger, Karl‐Heinz Ernst, Irena G. Stará, José Ángel Martín‐Gago, Ivo Starý, Leonhard Grill, Pavel Jelínek

**Affiliations:** ^1^ Institute of Physics Czech Academy of Sciences Cukrovarnická 10 16200 Prague 6 Czech Republic; ^2^ Faculty of Nuclear Sciences and Physical Engineering Czech Technical University in Prague Břehová 78/7 11519 Prague 1 Czech Republic; ^3^ Department of Physical Chemistry University of Graz Heinrichstraße 28 8010 Graz Austria; ^4^ Institute of Organic Chemistry and Biochemistry Czech Academy of Sciences Flemingovo nám. 2 16610 Prague 6 Czech Republic; ^5^ Instituto de Ciencia de Materiales de Madrid CSIC Cantoblanco 28049 Madrid Spain; ^6^ Regional Centre of Advanced Technologies and Materials Czech Advanced Technology and Research Institute (CATRIN) Palacký University 78371 Olomouc Czech Republic.; ^7^ Empa Swiss Federal Laboratories for Materials Science and Technology Überlandstrasse 129 8600 Dübendorf Switzerland

**Keywords:** azide, metal surface, nitrene, photochemistry, scanning probe microscopy

## Abstract

Nitrenes are known as key intermediates in various chemical reactions. Nitrene transfer reactions are particularly effective for synthesizing nitrogen‐containing compounds, where metal catalysts play a crucial role in controlling nitrene reactivity and selectivity. In this study, we demonstrate the formation of a stable surface‐supported dinitrene on Au(111) through UV irradiation of its diazide precursor, characterized by scanning probe techniques. The photoreaction mechanism is elucidated with wavelength‐dependent experiments and time‐dependent density functional theory calculations. Our findings present the first real‐space visualization of a metal nitrene adsorbed on a surface, highlighting its potential in catalysis and surface functionalization.

## Graphical abstract

In this communication we report the formation of a stable, surface‐supported dinitrene achieved through the photolysis of a diazide precursor on an Au(111) metal substrate. We combine high‐resolution scanning probe techniques with theoretical calculations, providing the first real‐space imaging of a metal nitrene adsorbed on a surface and exploring the associated photoreaction mechanism.

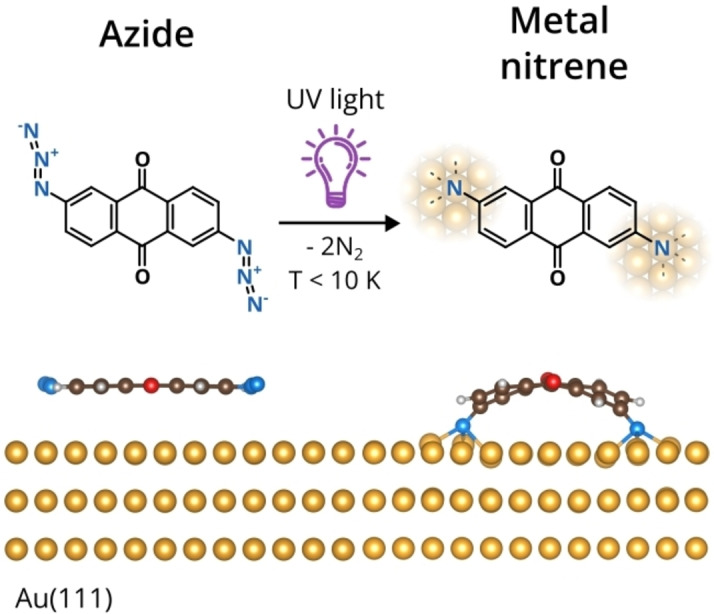

Nitrenes are highly reactive and short‐lived species, playing an important role as intermediates for a plethora of chemical reactions.[^1^, ^2^] In the last few years, there was a booming interest in skeletal editing methods, where nitrenes are often involved in N‐insertion processes.[^3^, ^4^, ^5^] Furthermore, the use of nitrene transfer reactions involving metal‐nitrenoid intermediates represents an efficient strategy for the synthesis of N‐containing compounds.^[6]^ In such reactions, the first step is the formation of a transition metal (TM) nitrene complex (see Scheme 1), which can further react towards the formation of new C−N bonds, e.g. through aziridination or C−H insertion. Several TMs including coinage metals have been reported to efficiently tame the nitrene reactivity, influencing its chemo‐ and enantioselectivity and catalyzing the reactions.[^7^, ^8^] In this regard, the stabilization of metal‐nitrene complexes allows to follow the stepwise process, study the structure of the intermediate and understand the mechanisms of nitrene transfer reactions. Nowadays nitrenes are routinely obtained from azides via photochemical, thermal, or metal‐mediated routes, with the elimination of dinitrogen as a side product.[^9^, ^10^, ^11^] The photochemistry of azides has been thoroughly investigated:[^12^, ^13^, ^14^, ^15^, ^16^] namely, singlet arylnitrenes can go through either a rearrangement or intersystem crossing, in the latter case leading to a triplet nitrene that can dimerize forming diazo compounds.^[17]^ Recently, a crystalline nitrene was synthetized by azide photolysis, presenting extraordinary stability.^[18]^

**Scheme 1:**
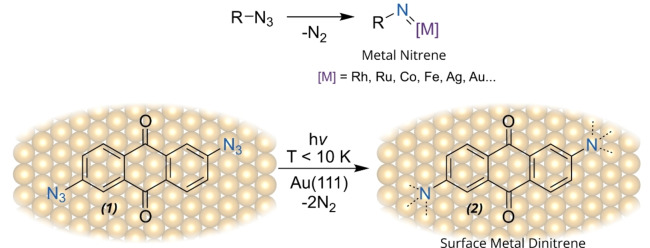
Top panel: nitrogen extrusion from azides leading to metal nitrene intermediates in solution chemistry. Bottom panel: in this paper we study the 2,6‐diazidoanthracene‐9,10‐dione precursor **1** and respective photogenerated dinitrene **2** stabilized on the Au(111) surface.

Within this framework, a known successful method to study reactive compounds such as azides (and the respective intermediates like nitrenes) is on‐surface chemistry under ultra‐high vacuum (UHV) conditions by means of scanning probe microscopy at cryogenic temperatures, as widely demonstrated for open‐shell carbon‐based nanostructures.^[19]^ Organic azides on surfaces have been mainly studied as precursors of the azide‐alkyne Huisgen cycloaddition reaction.[^20^, ^21^, ^22^] In the context of on‐surface chemistry, the use of light or tip manipulation as a stimulus offers a higher control compared to thermal‐assisted reactions, opening the possibility for low‐temperature reactions and thus facilitating the stabilization and study of reactive intermediates.[^23^, ^24^, ^25^] While the role of carbene ligands in surface chemistry has been explored in the last years,^[26]^ and surface metal carbenes have been studied at the nanoscale by scanning probe techniques,[^23^, ^27^, ^28^, ^29^] this characterization is still lacking for nitrenes due to their elusive nature.[^30^, ^31^, ^32^]

Here, we report a comprehensive scanning tunneling microscopy (STM), non‐contact atomic force microscopy (ncAFM), and computational study of a diazido anthraquinone molecule **1** and its photochemical activity on a Au(111) substrate, leading to the formation of a stable surface‐supported dinitrene **2** (see Scheme 1). To this aim, we sublimed **1** on a Au(111) surface under UHV conditions, revealing the growth of large islands. Subsequently, upon UV irradiation, we observe the selective transformation into a dinitrene anthraquinone **2** stabilized by the underlying Au(111). Ultimately, to rationalize the mechanism triggering the photoactivation of **1** and the role of the metal substrate, we performed wavelength‐dependence experiments, supported by time‐dependent density functional theory calculations (TD‐DFT). Altogether, we report the real‐space visualization of a metal nitrene adsorbed on a surface through a light induced reaction of azide functional groups, with prospects in the catalytic synthesis of N‐containing compounds or surface functionalization through strongly interacting molecular compounds.

Compound **1** was synthesized following the route described in Scheme S1.^[33]^ It was designed to expect flat adsorption on the surface, allowing SPM characterization, and its symmetry should lead to unambiguous adsorption mode. Furthermore, it proved to be stable enough to survive all the purification steps and thermal sublimation in UHV. After the solution synthesis, we studied the thermal stability of **1**: at 185 °C the molecules decompose, as visible in the supplementary video. The limited thermal stability of **1** calls for different reaction stimulus, and in this regard, photochemistry offers a convenient option to carry out reactions at room temperature (RT) or even at cryogenic temperatures and on a large scale. Therefore, we recorded the UV/Vis absorption spectrum of **1** in dichloromethane (DCM) solution (Figure S1), which shows a first absorption band centered around 350 nm, followed by more intense bands at higher energies (ca. 310 nm and 275 nm). Next, the molecular precursor **1** was sublimed at 135 °C on a Au(111) crystal kept at RT. After sublimation, we observe large and homogeneous islands extended throughout the surface, as illustrated in Figure 1a. Precursor **1** self‐assembles forming closed‐packed islands, exhibiting unit cell parameters of a=8.2 Å, b=12.8 Å and $\alpha =75^{\circ }$ (see Figure 1b). Organic azides are typically very reactive and after sublimation onto a coinage metal surface they immediately decompose.[^20^, ^30^] In order to confirm that **1** remains intact after deposition on Au(111), we performed ncAFM experiments. Figure 1c shows a high‐resolution ncAFM image where, importantly, all molecules remain intact after sublimation at RT, in contrast to previous works on organic azides on surfaces.^[30]^ The azido groups from adjacent transversal molecules are observed as two bright protrusions in between the molecules, suggesting intermolecular interactions. One of the main resonance forms of azido groups contains one nitrogen atom positively charged followed by another one negatively charged, as exemplified in Figure 1d for **1**. We can assume that these charges are retained on the surface, as visible in the comparison of calculated electrostatic maps of **1** in gas phase and on the Au(111) surface (Figure S2). Thus, we rationalize the molecular self‐assembly with an interplay between electrostatic interactions among azido groups and O⋅⋅⋅H hydrogen bonds (see electrostatic potential maps in Figure 1e).^[34]^ Due to the prochirality of **1**, we observe two chiral domains on the surface (see Figure S3), which can coexist on the same island. To investigate the molecule‐substrate interaction, we conducted force‐distance spectroscopic (Δf(Z)) measurements on different sites of the molecule and on Au(111) (see Figure S4) revealing an adsorption height of approximately 3 Å and confirming its planarity. This is in good agreement with the calculated DFT‐optimized geometry of the single neutral molecule **1** on Au(111), showing a molecule‐surface distance of 3.2 Å, compatible with a weak physisorption. Therefore, the electronic structure of **1** is only slightly perturbed by the surface, as shown by the calculated projected density of states (PDOS) in Figure S4 for both the free‐standing and adsorbed molecule.

**Figure 1:**
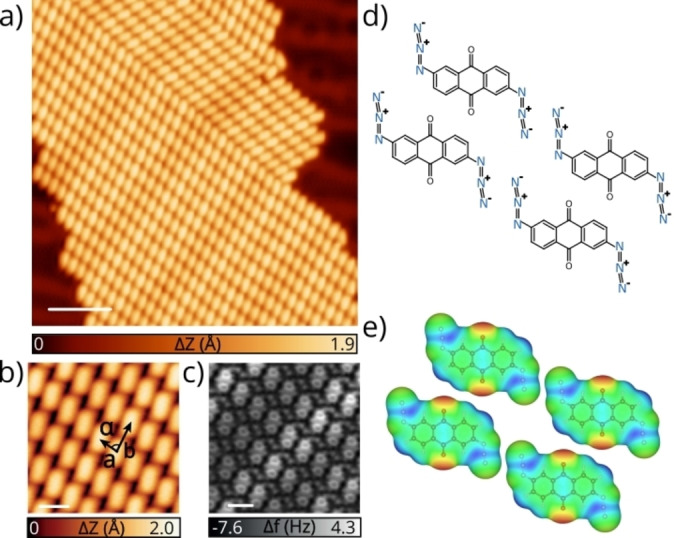
Structural characterization of diazide **1** on Au(111). a) Overview STM image of a closed‐packed island of **1** on Au(111). (*V*_b_=315 mV, *I*_t_=50 pA and scale bar=5 nm). b) Zoom‐in STM image showing the unit cells parameters. (*V*_b_=50 mV, *I*_t_=70 pA and scale bar=1 nm). c) NcAFM image of b). (*V*_b_=1 mV, scale bar=1 nm). d) Chemical sketch of the self‐assembly of **1** on Au(111). e) Calculated electrostatic potential surface of **1** in gas phase showing the charge distributions and the proposed intermolecular interaction.

Having confirmed that **1** adsorbs intact and planar on Au(111), we can investigate its photoreactivity, aiming at the generation of stabilized dinitrenes. We illuminated the sample at low temperature (T<10 K) with monochromatic UV light (λ=266 nm or 360 nm) targeting the excitation corresponding to the two absorption bands in the UV/Vis spectrum of **1** (Figure S1). In both cases we observe a clear change in the STM features after few minutes of illumination (see Figure S5), where each molecule is observed with the shape of three lobes, in contrast to diazide **1** which is essentially featureless. Figure 2a shows the same molecular island before and after 20 minutes of UV irradiation, imaged at the same bias voltage, where all molecules in the island show the same new features in the STM contrast. We investigated the chemical changes in **1** after illumination by ncAFM measurements. While the intact precursor lies flat and interacts weakly with the Au(111) (see simulated and experimental ncAFM in Figure 2b and calculated adsorption geometry in Figure 2c), the scenario changes after irradiation. As shown in Figure 2d, we cannot resolve the entire backbone of the molecule anymore in ncAFM, as only the central ring is visible. We assign this new product to dinitrene **2** (see Scheme 1), which is bent towards the Au(111) due to its high reactivity. The comparison between the simulated ncAFM image of **2** and the experiments (Figure 2d) validates our hypothesis, thus we can conclude that the illumination of **1** induces the release of two molecular N_2_ and the formation of the surface‐supported dinitrene. The photogenerated product **2** shows a new conformation, suggesting a strong interaction with the surface inducing a nonplanar geometry, as corroborated in the DFT optimized geometry of **2** on Au(111) (Figure 2e). The calculations show an important interaction between the nitrene and the surface: the change in the adsorption geometry is accompanied by a charge redistribution, consisting in an accumulation of electron density around the N atoms, each one interacting with three underlying gold atoms, as shown in Figure S6. The strong interaction between the nitrene and the surface quenches its reactivity, similarly to carbenes on Ag(111),^[23]^ making it stable enough to be imaged. We highlight the high yield of the reaction and its selectivity: mainly product **2** is observed (we determined a yield of more than 90 %), and very few molecules present an intermediate mononitrene.

**Figure 2:**
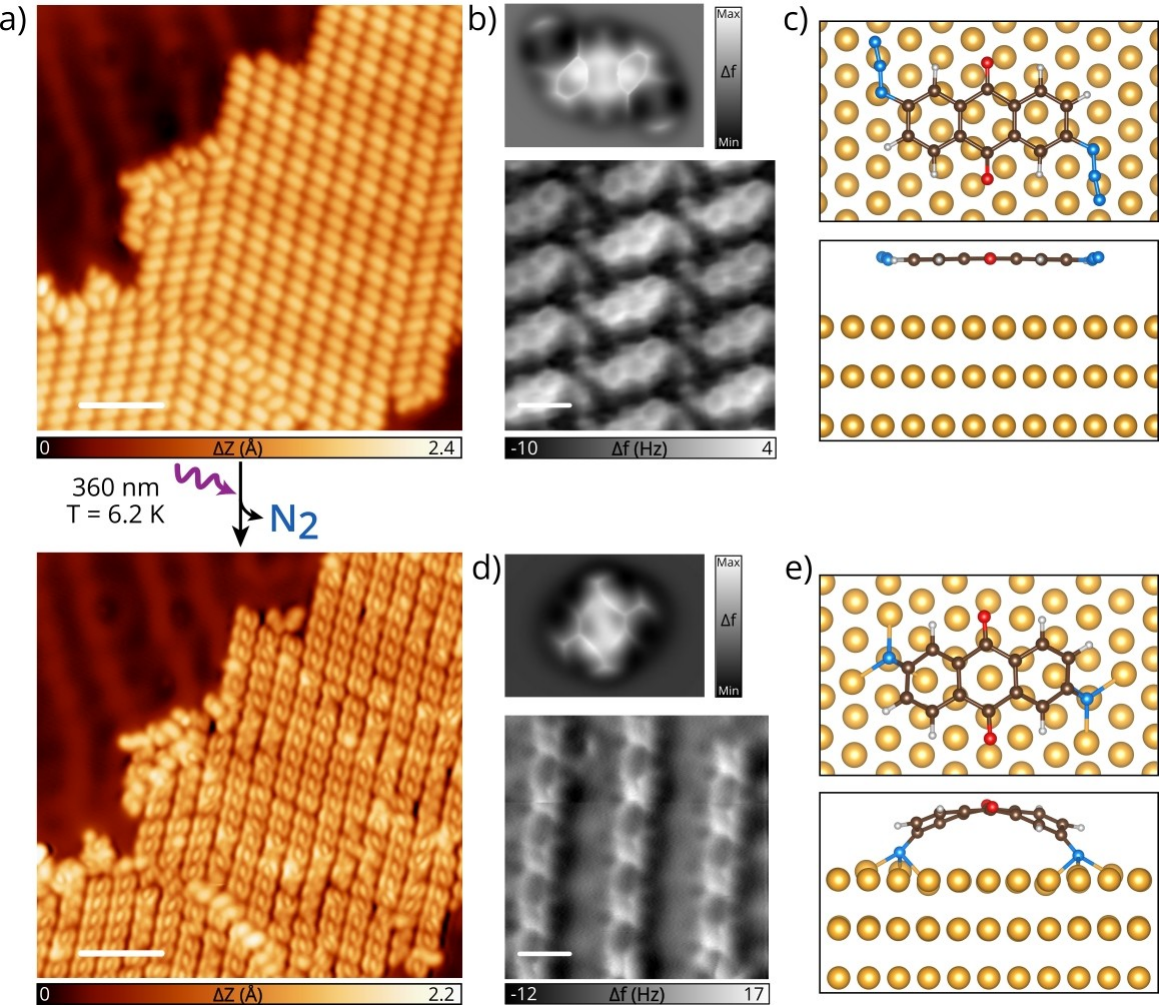
Light‐induced generation of dinitrene **2** from azide **1**. a) Overview STM image of the same molecular island of **1** on Au(111) before and after illumination at 360 nm for 20 minutes (*V*_b_=200 mV, *I*_t_,=150 pA and scale bar=4.2 nm for both images). b) Simulated (top panel) and experimental (bottom panel) ncAFM image (*V*_b_=2 mV and scale bar=7 Å) of **1** on Au(111). c) Top and side view of the calculated adsorption geometry of **1** on Au(111). d) Simulated (top panel) and experimental (bottom panel) ncAFM image of **2** on Au(111) (*V*_b_=1 mV and scale bar=7 Å). e) Top and side view of the calculated adsorption geometry of **2** on Au(111).

The generation of the dinitrene species **2** can also be followed step‐by‐step by local tip manipulation. In this case, we observe first the formation of the intermediate mononitrene and subsequent formation of **2**. A detailed description of the process is depicted in Figure S7. Hence, we demonstrated the formation of a nitrene on a metal surface either locally by stepwise tip manipulation, or on larger areas via UV irradiation. After forming **2** at LT, we examined its reactivity upon thermal activation. Annealing at 240 °C post‐illumination leads to the hydrogenation of the dinitrene, yielding diamino anthraquinone (**3**) along with other disordered structures (see Figure S8). Product **3** forms hydrogen‐bonded 1D assemblies.

It is important to mention that thermal activation of **1** on Au(111) at 240 °C results in the formation of various compounds and disordered dendritic structures. As shown in Figure S9, we investigated the chemical structure of the products by STM and ncAFM, revealing a mixture of different compounds where the majority of the molecules have lost N_2_. We performed quantum mechanics/molecular mechanics (QM/MM) calculations to rationalize the mechanism, which is mediated by single Au adatoms[^35^, ^36^] inducing the cleavage of the N=N bond, see Figure S10. Nevertheless, none of the products observed after annealing corresponds to the dinitrene anthraquinone **2**, while the light‐induced reaction shows a high selectivity and yield toward the formation of **2** and opens a new reaction pathway to selectively form product **3** (Figure S8). Thus, the loss of molecular nitrogen towards the formation of a stable nitrene on a surface is not accessible by thermal activation.

To investigate the mechanistic aspects behind the processes involved in the photolytic N=N bond cleavage in **1** (red bond in the inset of Figure 3e), including the role of the metallic substrate, we probed the light response of **1** after illuminating at three selected wavelengths (266 nm, 360 nm and 450 nm) rationalizing the results with TD‐DFT calculations, as summarized in Figure 3. The experimental and calculated absorption spectra in DCM solvent, together with a schematic representation of the vertical excited states, are displayed in Figure 3a,b. The three wavelengths employed in the experiments are also highlighted. Note that from the calculated absorption spectrum (Table S1), the first excited state (S_1_) is spectroscopically dark and is dominated by a *n*π* transition from an occupied orbital mainly localized on the carbonyl lone pairs (*n*_CO_) to a virtual delocalized π orbital (see Figure S11). The intense absorption peaks correspond to higher excited states (respectively S_4_, S_8_ and S_9_), dominated by transitions of ππ* character, namely involving π orbitals delocalized on the whole conjugated system. We chose to illuminate 360 nm and 266 nm in order to match two different intense absorption bands in solution and 450 nm, which is below the molecular absorption threshold. Since all the experiments are carried out on a metallic surface, we must consider two possible excitation mechanisms: direct intramolecular excitation and hot electron attachment (HEA) from the metallic surface, where an excited photoelectron is attached to an unoccupied molecular orbital.[^37^, ^38^]

**Figure 3:**
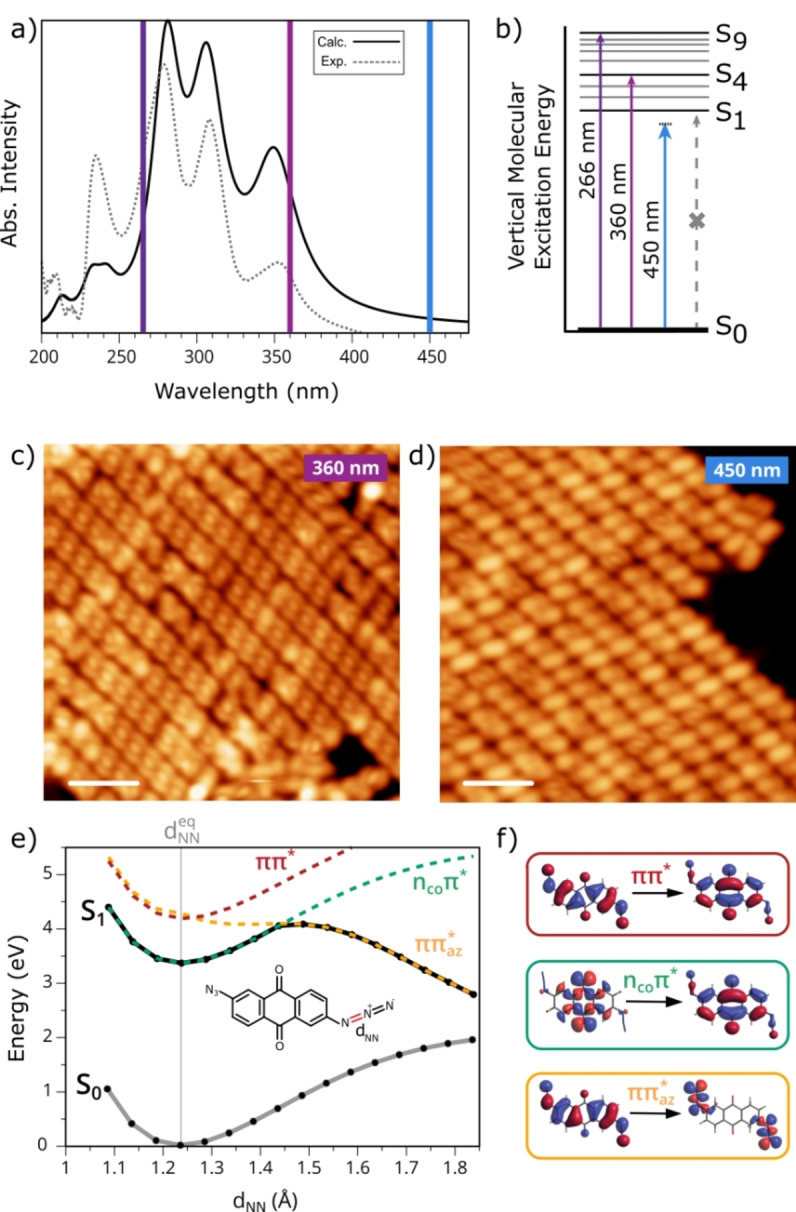
Insight into the light‐induced reaction of **1**. a) Calculated (solid line, Lorentzian broadening, rigid shift of −0.55 eV) and experimental (dashed) absorption spectra of **1** in DCM solvent, where the three used wavelengths are highlighted. b) Scheme of vertical excited states c) STM image of a molecular island after illumination at 360 nm (*V*_b_=100 mV and I_t_=100 pA, scale bar=3 nm). d) STM image of a molecular island after illumination at 450 nm (*V*_b_=100 mV and I_t_=105 pA, scale bar=3 nm). e) Calculated potential energy curves upon elongation of the N=N bond (red in the inset): ground state S_0_ (solid grey curve) and first excited state S_1_ (solid black curve). The colored dashed curves follow the energy evolution of the excited state dominated by the transition indicated with the same color (the states at the equilibrium geometry $d_{NN}^{eq}$ correspond to S_1_, S_4_, and S_5_). f) Molecular orbitals involved in the relevant transitions. We can see how upon N=N elongation the transition from a π orbital to an orbital localized on the azido moiety (yellow) tends to lower its energy, giving S_1_ a dissociative character at longer d_NN_ lengths.

As shown above, illumination at 360 nm and 266 nm leads to the formation of the same product with a very high yield (>90 %) after only a few minutes of illumination. When illuminating at 450 nm, no photoinduced processes should occur if we assume a negligible role from the substrate. However, we observe a partial photoconversion of **1** into **2**, with a yield around 26 % (see Figure 3d and S12). Note that in order to reliably compare the results obtained at different wavelengths, we irradiated the sample at 360 nm and 450 nm with the same optical power, measured in a parallel setup (details in the methods) for 1 hour, the respective STM images after illumination are visible in Figure 3c,d (images before illumination for both samples are shown in Figure S13 as a comparison).

To rationalize the experimental results, we performed calculations showing the evolution of the excited states energies following the elongation of the N=N bond cleaved in the photoinduced process. As previously discussed, the excitation at 360 nm pumps the molecular system in the S_4_ state (Figure 3b and 3e, red line at $d_{NN}^{eq}$ , and Figure 3f red). At the molecular equilibrium geometry (at which the photoexcitation occurs), the state S_5_ (Table S1, Figure 3e yellow line at $d_{NN}^{eq}$ and Figure 3f yellow) is nearby in energy (approx. 0.1 eV above S_4_) and dominated by the ππ_az_* transition from a π occupied orbital to a virtual one localized on the azide fragments (π_*az*_). Interestingly, upon minimal bond elongation (as low as 0.05 Å), the ππ_az_* state crosses below ππ* (in energy) with almost no barrier and, due to its strong dissociative character along d_NN_ bond length coordinate (no minimum found), its energy drops until becoming the lowest excited state at only 0.3 Å of elongation (i.e. at d_NN_∼1.5 Å). Hence, following photoexcitation promoting the system to the active S_4_ ππ* state (at $d_{NN}^{eq}$ ), a fast conversion to the dissociative ππ_az_* state can occur, leading to an efficient N=N dissociation. This is in line with the observed high yield and fast process of the present experiments (with λ=360 nm) and with previous studies of the photophysical processes preceding azides photodissociation.[^39^, ^40^, ^41^, ^42^, ^43^]

As another deactivation path after photoexcitation, the molecule can reach S_1_ state via internal conversion (Kasha rule).^[44]^ Upon bond elongation (about 0.3 Å), S_1_ acquires ππ_*az*_ * dissociative character (Figure 3f yellow, Table S1), suggesting that also in this case the dissociation can occur along the black curve in Figure 3e, provided the overcoming of an energetic barrier that lowers the overall efficiency of this process. In the case of excitation at 266 nm, similar experimental results suggest similar processes that evolve starting from the higher excited state S_9_.

Lastly, we can interpret the non‐negligible yield after irradiation at 450 nm, that is below the energy necessary to promote the molecule to the first excited state via photoexcitation, with a contribution from the metallic substrate through HEA. Thus, this mechanism is also involved in the process, although in a less efficient way, resulting in a lower photoconversion yield. A similar effect was observed in the photodecomposition of azides in the presence of a Pd catalyst, extending their photoreactivity to longer wavelengths.^[45]^

We can deduce that the predominant process taking place is molecular photoexcitation, due to the low interaction between the molecule and the metallic substrate. However, the contribution from HEA mechanism is not negligible and thanks to the synergy between the two processes occurring in the light‐mediated reaction, we observe an efficient photochemical reaction on Au(111) that generates dinitrenes with high yield and selectivity.

We conclude that in the two proposed reaction mechanisms, the photochemical reaction is driven by the presence of the dissociative excited state localized on the azido moiety (yellow state in Figure 3f), leading to the N=N cleavage and the consequent nitrene formation and surface‐mediated stabilization.

In summary, we report the generation and characterization of a dinitrene on a metal surface, synthetized by photolysis of its diazide precursor. While diazide is only physisorbed on Au(111), the photogenerated dinitrene interacts strongly with the substrate, that quenches its reactivity, allowing the characterization by scanning probe techniques. Our findings pave the way for understanding and tuning (di)nitrene reactivity on surfaces, offering new prospects in nitrene surface chemistry and surface functionalization. Furthermore, we performed wavelength‐dependent experiments and TD‐DFT calculation, rationalizing the molecular states involved in the photodissociation, highlighting a synergy between molecular photoexcitation and hot electron attachment.

## Conflict of Interests

The authors declare no conflict of interest.

## Supporting information

As a service to our authors and readers, this journal provides supporting information supplied by the authors. Such materials are peer reviewed and may be re‐organized for online delivery, but are not copy‐edited or typeset. Technical support issues arising from supporting information (other than missing files) should be addressed to the authors.

Supporting Information

Supporting Information

## Data Availability

The data that support the findings of this study are available from the corresponding author upon reasonable request.
