# Supplementary material for: Photogeneration and Visualization of a Surface‐Stabilized Dinitrene
Source: Angew Chem Int Ed Engl. 2025 Mar 6;64(20):e202502640. doi: 10.1002/anie.202502640 (PMC12070354; doi:10.1002/anie.202502640)
Supplement: Supplementary file 2 — Supporting Information [file ANIE-64-e202502640-s002.pdf]

Angewandte Chemie
Eine Zeitschrift der Gesellschaft Deutscher Chemiker

Supporting Information

Photogeneration and Visualization of a Surface-Stabilized Dinitrene

F. Frezza, A. Sánchez-Grande, S. Canola, C. Nacci, J. Klívar, P. Mutombo, Q. Chen, J. M. Gómez-Fernandez, C. Sánchez-Sánchez, J. Berger, K.-H. Ernst, I. G. Stará, J. Á. Martín-Gago*, I. Starý*, L. Grill*, P. Jelínek**

Supporting Information

Photogeneration and visualization of a surface-stabilized dinitrene

Federico Frezza,[1,2] Ana Sánchez-Grande,[1] Sofia Canola,[1] Christophe Nacci,[3] Jiří Klívar,[4] Pingo Mutombo,[1] Qifan Chen,[1] José María Gómez,[5] Carlos Sánchez-Sánchez, [5] Jan Berger[1,6], Karl-Heinz Ernst,[1] Irena G. Stará,[4] José Angel Martín-Gago,*[5] Ivo Starý,*[4] Leonhard Grill,*[3] Pavel Jelínek*[1,6]*

[1] Institute of Physics, Czech Academy of Sciences. Cukrovarnická 10, 16200 Prague 6, Czech Republic.

[2] Faculty of Nuclear Sciences and Physical Engineering, Czech Technical University in Prague, Břehová 78/7, 11519 Prague 1, Czech Republic

[3] Department of Physical Chemistry, University of Graz, 8010 Graz, Austria

[4] Institute of Organic Chemistry and Biochemistry Czech Academy of Sciences. Flemingovo nám. 2, 16610 Prague 6, Czech Republic.

[5] Instituto de Ciencia de Materiales de Madrid, CSIC, Cantoblanco, 28049 Madrid, Spain.

[6] Regional Centre of Advanced Technologies and Materials, Czech Advanced Technology and Research Institute (CATRIN), Palacký University Olomouc. 78371 Olomouc, Czech Republic.

Contents

Synthetic procedure 2
Methods 4
Figure S1**.** UV-Vis absorption spectrum of **1** in dichloromethane 6
Figure S2**.** Calculated electrostatic potential surfaces of **1** 6
Figure S3. Molecular chirality of **1** on Au(111) 7
Figure S4. Z spectroscopies and electronic structure of **1** on Au(111) 7
Figure S5. STM images after UV irradiation 8
Figure S6. Calculated charge transfer maps of **2** on Au(111) 8
Figure S7. Tip-induced formation of dinitrene **2** 9
Figure S8. STM images after annealing of **2** on Au(111) 10
Figure S9. STM images after annealing of **1** on Au(111) 10
Figure S10. QM/MM calculations of the azide dissociation on Au(111) 11
Table S1. Calculated vertical excited states 11
Figure S11. Molecular orbitals involved in the photoexcitation of **1** 12
Figure S12. STM images after illumination at 450 nm 12
Figure S13. STM images before and after illumination at 360 and 450 nm 13
References 14

Synthesis of 2,6-diazidoanthracene-9,10-dione (1)

2,6-Diazidoanthracene-9,10-dione **1** was prepared according to modified literature procedures.[1,2]

A solution of 2,6-diaminoanthracene-9,10-dione (1.191 g, 5.0 mmol) in acetic acid (9.2 mL) and sulphuric acid (4.3 mL) was cooled to 0 °C, and a solution of sodium nitrate (700 mg, 10.2 mmol, 2.0 equiv.) in water (5 mL) was added dropwise. Then an aqueous solution of urea was added and, after 10 min, followed by a slow addition of a solution of sodium azide (7.70 g, 118.5 mmol, 23.7 equiv.) in water (50 mL). The reaction mixture was left to warm to room temperature overnight. The mixture was poured into ice water (100 mL) and extracted with ethyl acetate (3×50 mL). The organic phases were combined and dried with anhydrous magnesium sulphate. The solvent was removed *in vacuo* and the reside was purified by flash column chromatography on silica gel (hexane – DCM 40:60, then chloroform), followed by another flash column chromatography on silica gel (chloroform) and crystallization from chloroform. Yellow small crystals were washed with excess of pentane to give diazide **1** (124 mg, 9%).

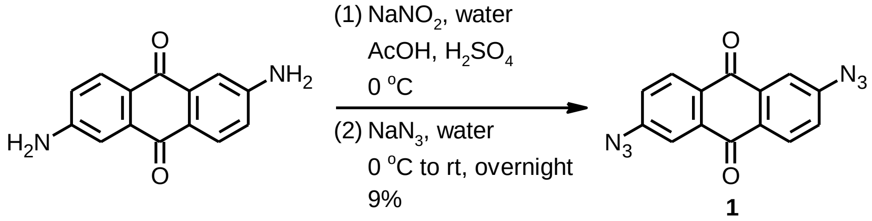

Scheme S1. Synthesis of 2,6-diazidoanthracene-9,10-dione **1**

M.p.: decomposition above 185 °C.

^{1}H NMR (400 MHz, CD_2Cl_2): 7.40 (dd, *J* = 8.3, 2.4 Hz, 2 H), 7.92 (d, *J* = 2.5 Hz, 2 H), 8.29 (d, *J* = 8.3 Hz, 2 H).

^{13}C NMR (101 MHz, CD_2Cl_2): 117.38 (2×CH), 124.82 (2×CH), 130.10 (2×CH), 130.56 (2×C_q), 135.78 (2×C_q), 147.35 (2×C_q), 181.67 (2×C_q).

IR ($CHCl_3$): 3074 vvw, 3062 vvw, 2120 s, 1675 m, 1591 s, 1575 w, 1486 vw, 1338 w, 1320 m, 1310 vs, 1280 w, 1247 w, 1170 w, 1134 vw, 1080 vw, 895 w, 879 w, 841 vw, 559 vw, 493 w cm^{-1}.

TOF EI MS: 262 (100), 234 (72), 206 (18), 178 (63), 151 (92), 117 (11), 89 (25), 62 (28).

HR TOF EI MS: calcd for $C_{14}H_6N_6O_2$ 290.0547, found 290.0543.

^{1}H-NMR of 1

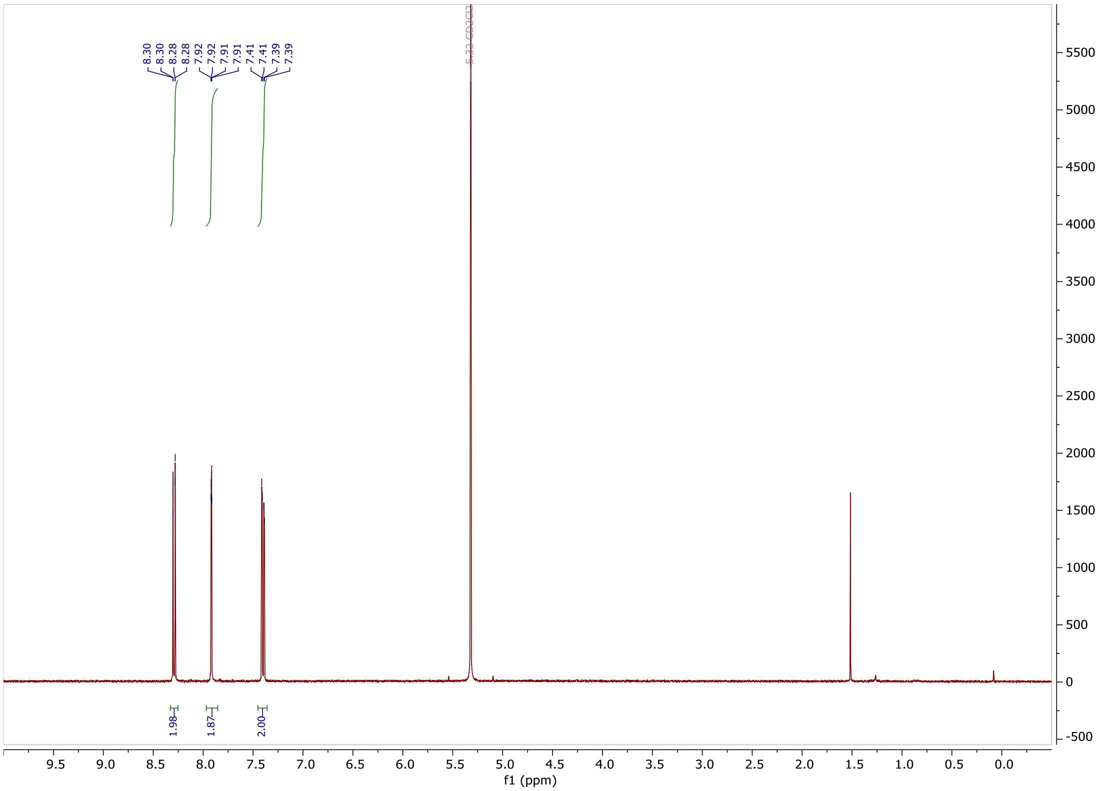

^{13}C-NMR of 1

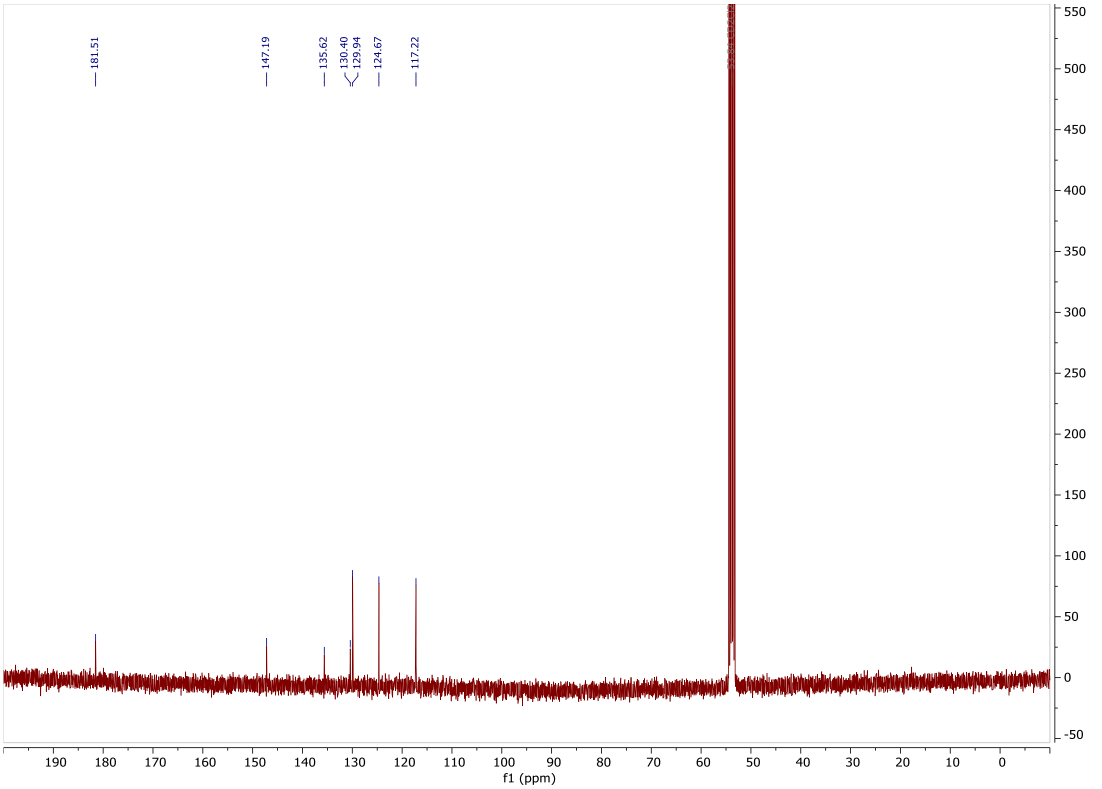

Methods

The experiments were performed in three different UHV chambers in Graz, Austria, and Prague and Olomouc, Czech Republic. The illumination experiments at different wavelength (266 nm, 360 and 450 nm) and the relative STM measurements were performed in Graz. NcAFM experiments were performed in Czech Republic. The sample preparation was the same in all cases.

Sample preparation

The Au(111) substrate (MaTeck GmbH) was cleaned by repeated cycles of Ar+ ion sputtering (E = 1 keV) and subsequent annealing to 740 K for 10 minutes. The molecular precursor were thermally sublimed from a Ta or quartz crucible onto the Au(111) surface kept at room temperature, sublimation temperature 130 – 145 °C.

Sample illumination

Samples were always illuminated in ultrahigh vacuum (UHV) through fused silica viewports. The samples were irradiated directly into the STM head, and the temperature during irradiation never exceeded 10 K. The following laser sources were employed: CryLas FQSS266-Q2 or CNI Laser MPL-F-266 (266 nm), Roithner RLTUVL-360-10-3 (360 nm) and Thorlabs CPS450 (450 nm).

To reliably compare the illumination at different wavelengths we regulated the power of our 360 nm and 450 nm sources to have the same optical power of 4.2 mW, measured with a power meter (Thorlabs PM100A) placed at a distance of 24 cm, replicating the experimental set-up.

STM and ncAFM experiments

Experiments were performed in custom-designed ultra-high vacuum systems (base pressure below 5 x 10^{-10} mbar) hosting a commercial low-temperature microscope with STM/AFM capabilities CreaTec GmbH. Unless otherwise noted, all STM images were taken in constant-current mode at a sample temperature of 5.0 K. Scanning parameters are specified in each figure caption. Non-contact AFM measurements were performed with Pt/Ir tip attached to a Qplus tuning fork sensor from Createc.[3] The tip was a posteriori functionalized by controlled adsorption of a single CO molecule at the tip apex from a previously CO-dosed surface.[4] The sensors were driven at their resonance frequency (30 kHz) with a constant amplitude of ~ 60/50 pm. The frequency shift from the resonance of the sensor (with the attached CO-functionalized tip) was recorded in a constant-height mode (Nanonis SPM for Createc GmbH). STM and ncAFM images were analyzed using WSxM.[5]

DFT calculations

Density Functional Theory calculations were performed using the GGA-PBE approximation of the exchange-correlation potential[6] as implemented in the Fritz-Haber Institute *Ab-Initio* Materials Simulation code (FHI-AIMS)[7] to study the electronic properties of the azide molecules at the Au(111) surface. The Tkachenko-Scheffler treatment of the Van der Waals interactions[8] was included in all the calculations. All the molecules were submitted to an atomic relaxation before placing them on the surface. This was done using the hybrid functional B3LYP (Becke, 3-parameter, Lee–Yang-Parr)[9,10]). The 'tight' atom-centered orbital bases of the FHI-AIMS code were employed to expand the Kohn–Sham wave functions.

The relaxed molecules were put at the 7x10 Au (111) surface made of 3 layers. Structural optimizations of all the atoms of the slabs were performed, except the bottom Au layer. The calculations were converged when the total energy difference and the remaining atomic forces were lower than 10^{-5} eV and 10^{-2} eV/Å, respectively. A single gamma point was used for the integration in the Brillouin zone. The electrostatic potential maps and the charge density difference were calculated to describe the charge redistribution within the molecules and at the interface between them and the surface.

AFM simulations

The AFM Simulations were carried up employing the ProbeParticle code,[11,12] which takes into account the van der Waals (vdW) and the electrostatic interactions between the CO tip and the surface, to compute the images. The van de Waals interactions, the electrostatic forces between the CO tip and the surface as well as the Pauli repulsion were determined from the Hartree potential and the total density obtained from the DFT calculations. The simulations were done using a lateral stiffness of (k)=0.25 N/m.

QM/MM calculations

For the QM/MM simulations, we have employed the Fireball/AMBER software.[13] The classical region covers part of metal Au(111) surface. The quantum region consists of the molecules and a single adatom with underlying metal cluster underneath. The QM region is treated at the BLYP-DFT level with the Fireball software,[14] a local-orbital DFT code that employs an optimized basis set of pseudo-atomic orbitals. The forces obtained from the QM region are managed by AMBER and integrated to derive the dynamics. We have employed a free molecular QM/MM dynamic at room temperature to check the thermal stability of the system. We use the Langevin thermostat implemented in AMBER to simulate the canonical ensemble.

Excited states dissociation curves

Calculations were done employing DFT method for the molecular electronic ground state and TD-DFT for the excited states. wB97X-D functional[15] and 6-31G* basis set were employed, in the implementation of Gaussian16 Rev. C.01 software.[16] The diazido-anthraquinone molecule (***1***, Scheme 1) has been optimized in its ground state in vacuo and subsequently its vertical excited states have been computed at the equilibrium geometry. To simulate the absorption spectrum, the calculation has been repeated including the dichloromethane as solvent via Polarizable-Continuum model (PCM);[17,18] the vertical transitions (stick spectrum) have been broadened with a Lorentzian line shape (half width half maximum 0.05 eV) and rigidly shifted of -0.55 eV for a better comparison with the experiment. The potential energy curves are computed starting from the ground state equilibrium geometry and elongating the cleaving N-N bond (step ±0.05 Å). For each point, the ground and lowest excited states are computed. The curve of each state is plotted as an energy difference between its energy at each point and that of the relaxed minimum on S_0.

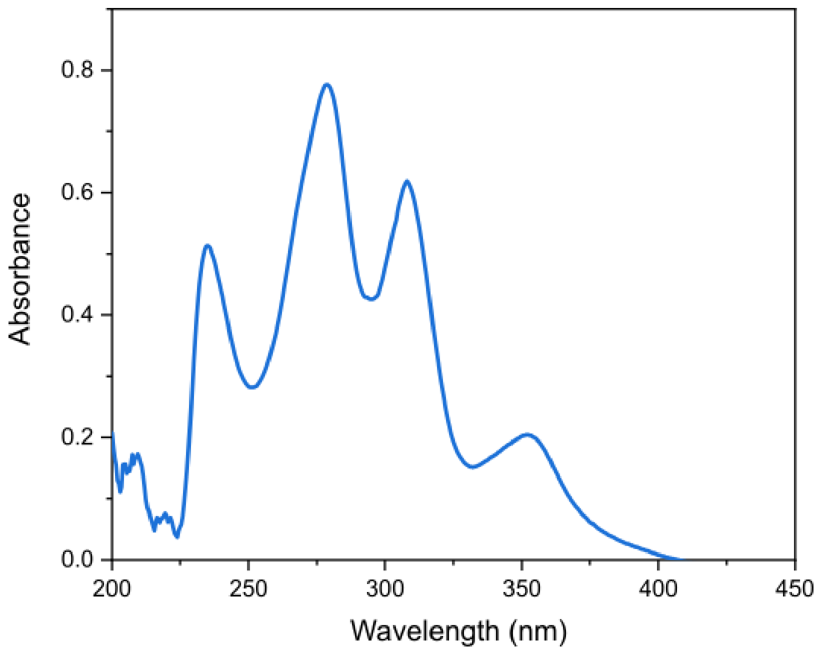

Figure S1. UV-Vis absorption spectrum of **1** in dichloromethane (DCM).

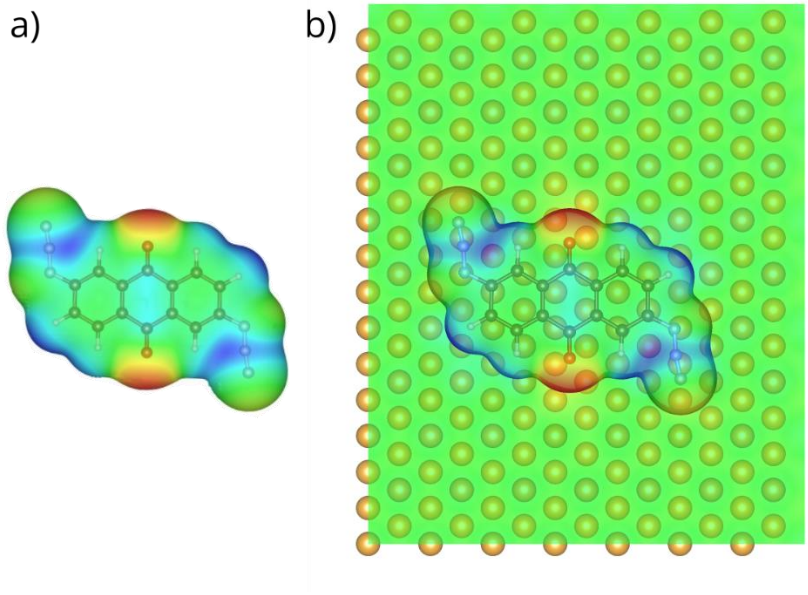

Figure S2. Calculated electrostatic potential surface of **1** a) in gas-phase and b) on Au(111). The charge distribution is retained on the surface.

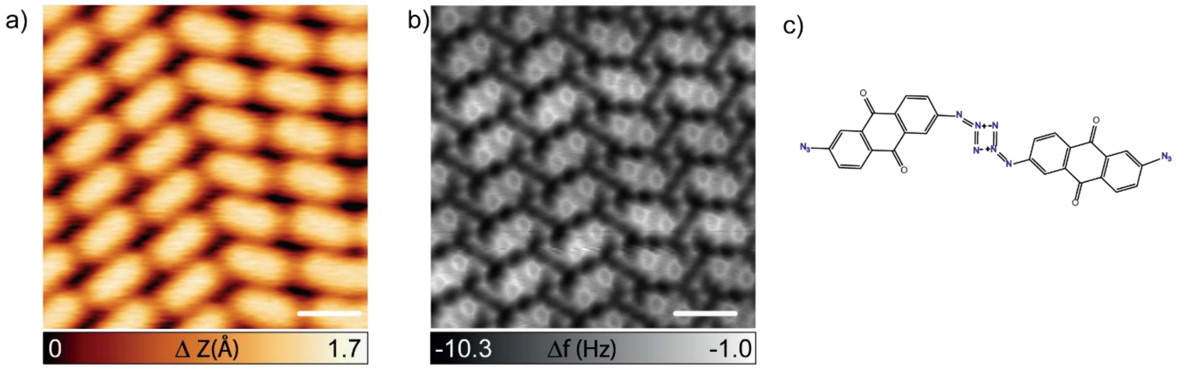

Figure S3. Molecular chirality of **1** on Au(111). a) STM (V_b = 50 mV, I_t, = 10 pA and scale bar = 1.2 nm). and b) nc-AFM (V_b = 3 mV, scale bar = 1.2 nm) images of two chiral domains. c) Chemical representation of the intermolecular interactions at the boundary of the domains.

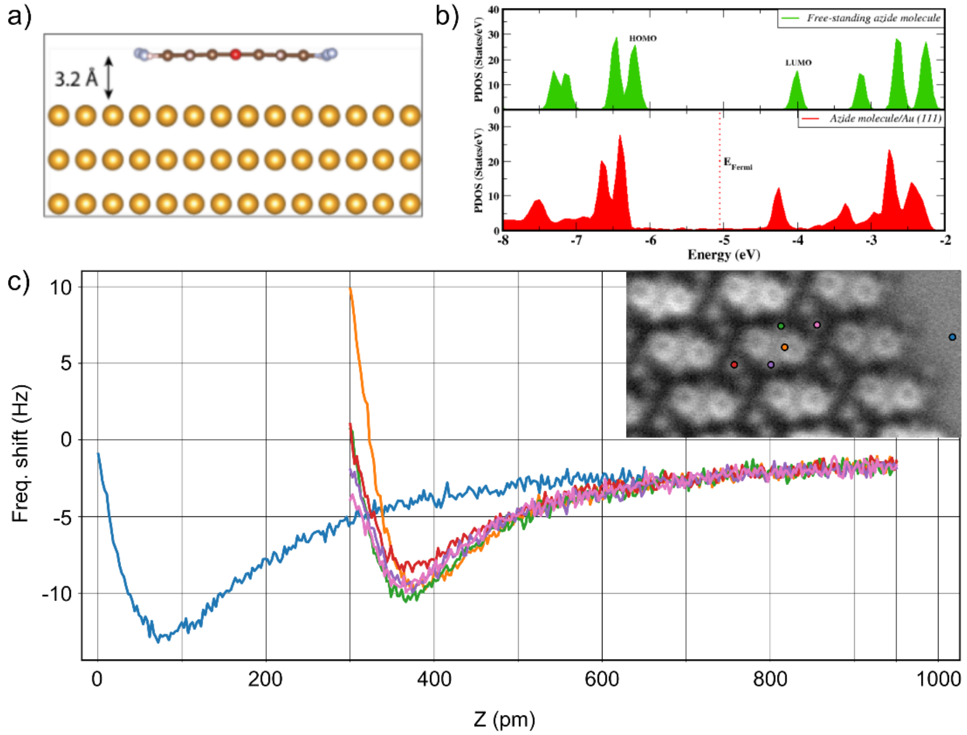

Figure S4. a) DFT-optimized geometry of ***1*** on Au(111), b) Calculated electronic structure of **1** in the free-standing case (green) and adsorbed on Au(111) (red). The respective projected densities of states (PDOS) show only negligible renormalization of the frontier molecular orbitals. c) Z spectroscopies on different sites of the molecule and on Au(111) relative to the ncAFM image of **1** on Au(111) in the inset, size 4.7 nm x 2.35 nm, V = 1 mV

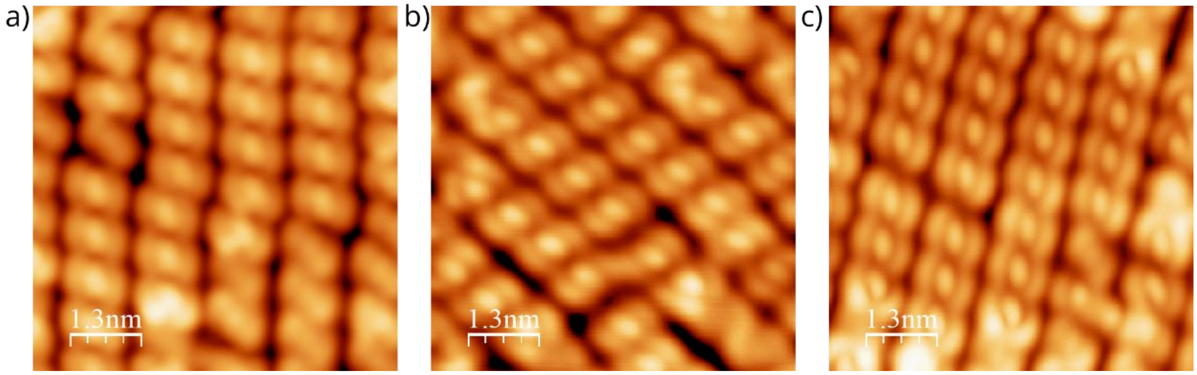

Figure S5. STM images after UV irradiation of **1** on Au(111) for a) 5 minutes at 266 nm, V = 100 mV, I = 15 pA b) 20 minutes at 266 nm in a different experimental setup, V = 100 mV, I = 125 pA, c) 20 minutes at 360 nm, V = 200 mV, I = 65 pA. In all cases we observe the three-lobes feature indicating the formation of the dinitrene **2**.

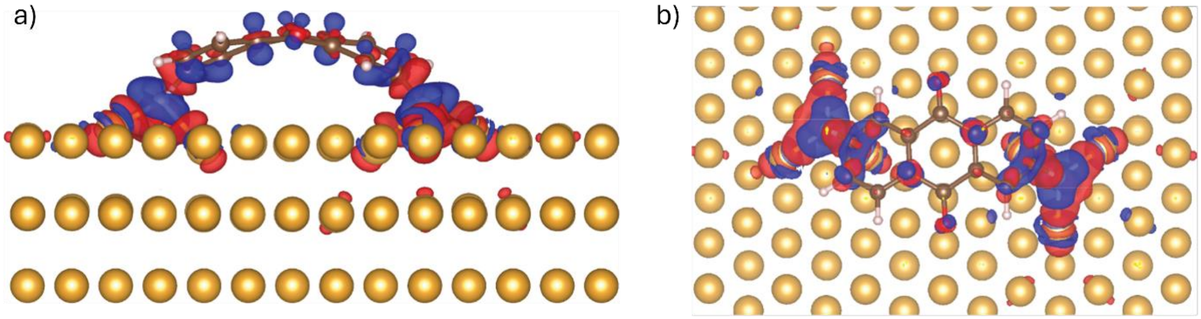

Figure S6. a) Side and b) top view of the optimized structure of the dinitrene **2** on Au(111) showing the calculated charge transfer. The blue and red clouds represent accumulation and depletion of electronic density, respectively. Isosurfaces 0.02 e/Å^3.

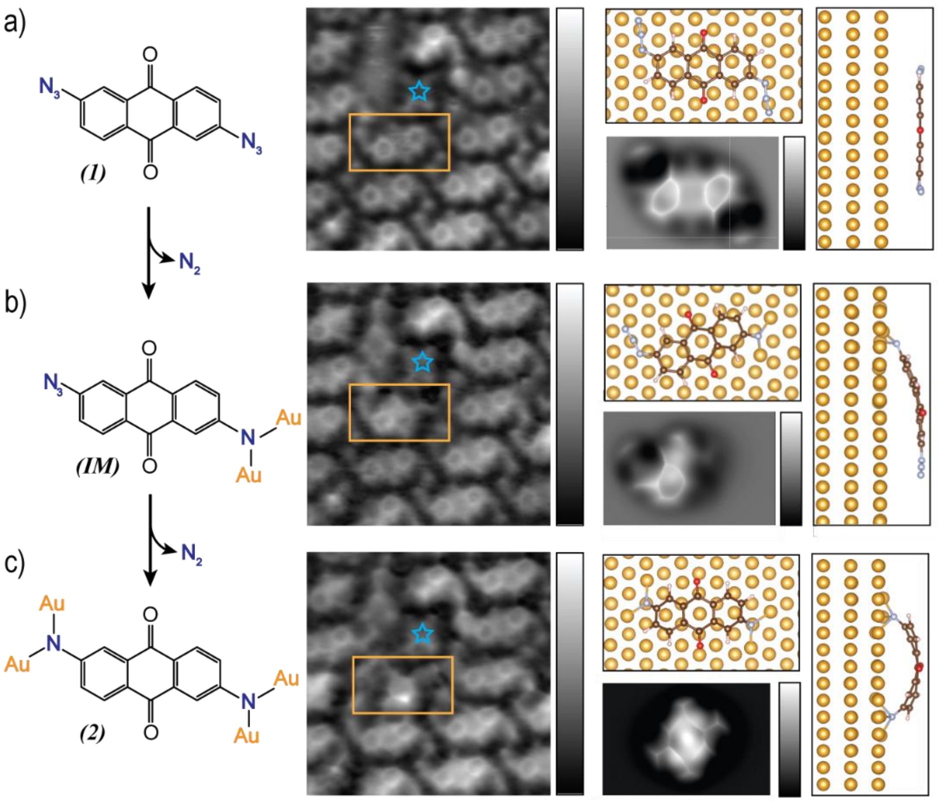

Figure S7. Tip-induced formation of dinitrene **2**. a) Chemical sketch of **1**, the nc-AFM image of a molecular island and the DFT calculations showing the geometry adsorption of **1** on Au(111), together with its corresponding simulated nc-AFM image. The orange square indicates the molecule that will be tip-manipulated toward the formation of **2** and the blue star is used as reference. b) The N=N cleavage was induced by placing the STM tip over the external ring of the anthraquinoid backbone and switching the feedback off at $V_b = 0.2$ V and $I_t = 5$ pA. From this set point, the bias voltage was increased to ≈ 1.8 V, observing a jump in the tunneling current indicating the formation of the intermediate mononitrene (**IM**) c) positioning the tip over the other external ring of the anthraquinoid backbone and repeating the manipulation event, the removal of other N_2 molecule and the formation of **2** is induced.

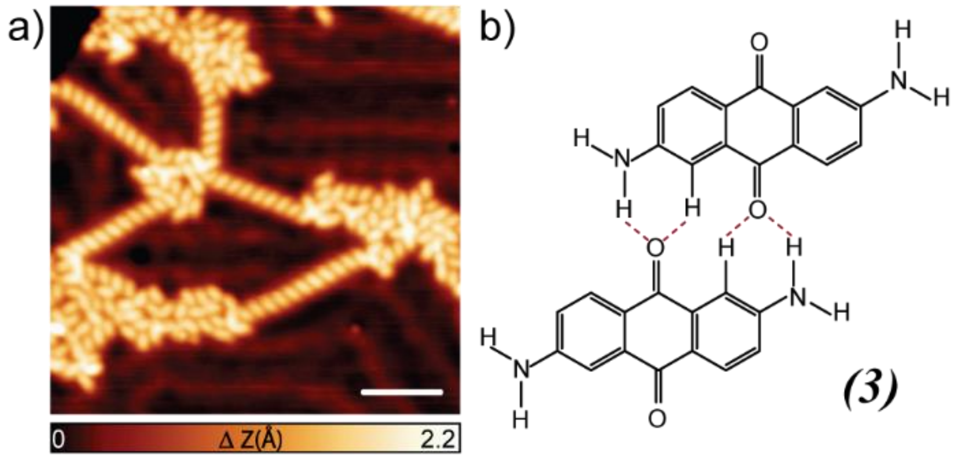

Figure S8. Annealing at 240 °C of dinitrene **2** on Au(111). a) STM image after annealing **2** to induce hydrogenation and subsequent formation of chains of 2,6-diaminoanthraquinone **3** (V_b= 60 mV and I_t = 30 pA, scale bar = 5 nm). b) Chemical scheme of a dimer of **3** stabilized by hydrogen bonds.

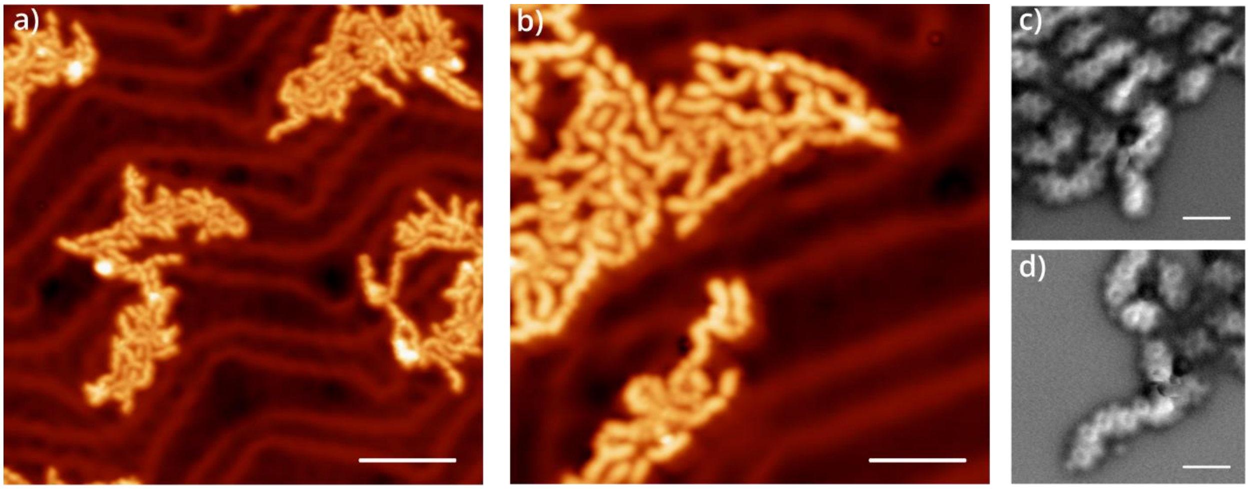

Figure S9. Annealing of **1** at 240 °C on Au(111). a) 50 mV, 20 pA, scale bar = 8 nm. b) 100mV, 20pA, scale bar = 4 nm, c,d) ncAFM images of the products, V = 1 mV, scalebar = 1 nm.

a) b)

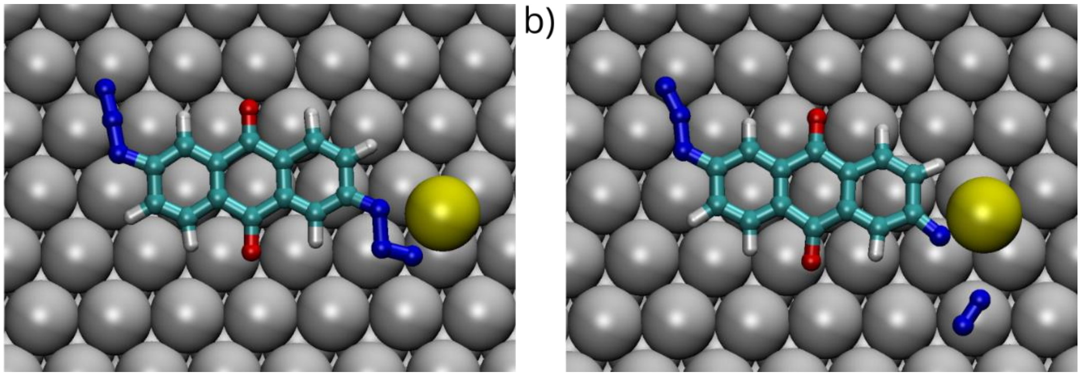

Figure S10. QM/MM calculations of the azide group dissociation in the presence of a Au adatom, showing a barrierless N=N cleavage at RT. Two snapshots of the simulation show the a) initial and b) final configuration. While in the initial configuration **1** is intact in the presence of a single Au adatom, in the final one the Au adatom forms a chemical bond with the N atom, and the N_2 molecule is released.

n. state	Exc./ eV (nm)	osc. st.	configuration
**S1**	**3.37 (368)**	**0.000**	**0.86 HOMO-2 → LUMO**
S2	3.63 (342)	0.000	0.78 HOMO-5 → LUMO
S3	3.78 (328)	0.000	0.91 HOMO → LUMO
**S4**	**4.19 (296)**	**0.261**	**0.67 HOMO-1 → LUMO**<br>**0.18 HOMO-4 → LUMO**
S5	4.28 (290)	0.000	0.44 HOMO → LUMO+2<br>0.40 HOMO-1 → LUMO+3
S6	4.28 (290)	0.001	0.40 HOMO-1 → LUMO+2<br>0.44 HOMO → LUMO+3
S7	4.49 (276)	0.000	0.71 HOMO-3 > LUMO
**S8**	**4.68 (265)**	**0.602**	**0.46 HOMO → LUMO+1**<br>**0.20 HOMO-1 → LUMO**<br>**0.18 HOMO-4 → LUMO**
**S9**	**5.10 (243)**	**0.623**	**0.56 HOMO-4 → LUMO**<br>**0.27 HOMO → LUMO+1**

Table S1. Relevant lowest vertical excited states (TD-wB97XD/6-31G*) at the ground state optimized geometry: order of state, excitation energy, oscillator strength and wavefunction composition (dominant configuration: molecular orbitals between which transition occurs and normalized weight coefficient).

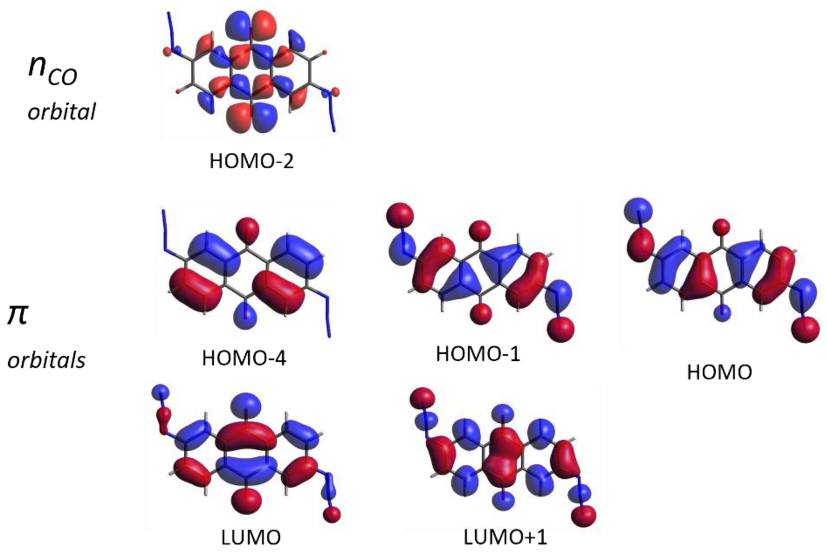

Figure S11. Molecular orbitals involved in the first (S1) and in the other active excited states (S4, S8, S9) of **1** (TD-wB97XD/6-31G*).

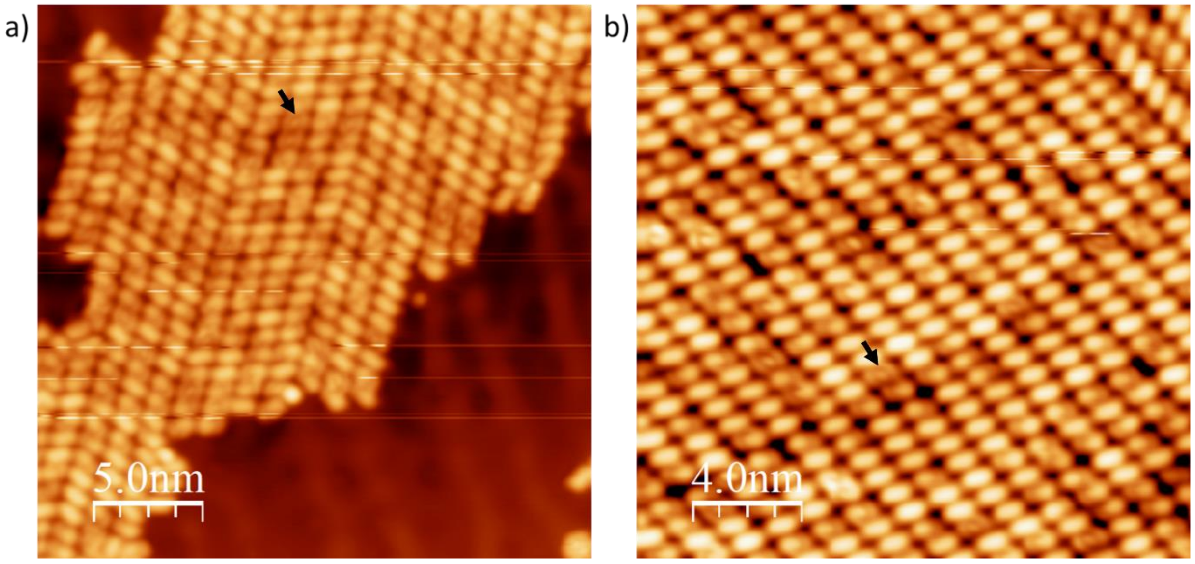

Figure S12. Molecular islands after illumination of **1** at 450 nm. a,b) V = 100 mV, I = 100 pA. Black arrows indicate one dinitrene **2** in both images.

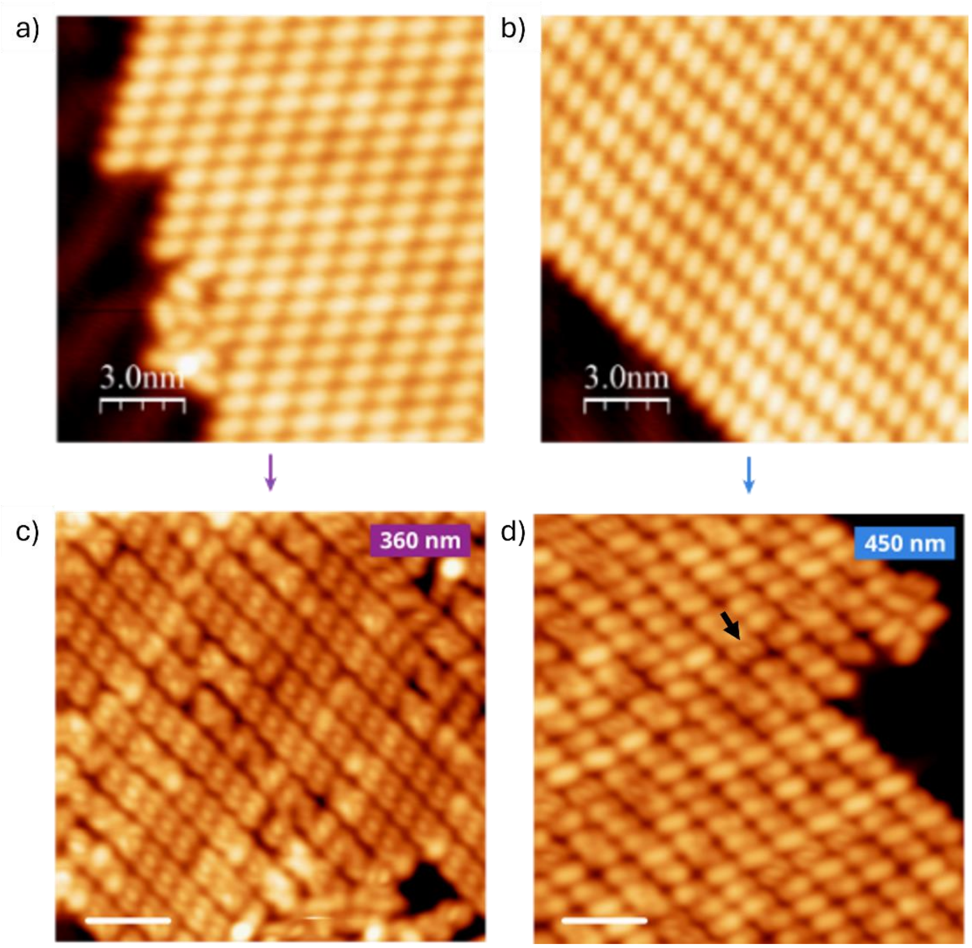

Figure S13. Islands of intact molecules before and after illumination of **1** at a) 360 and b) 450 nm, corresponding to c,d) the two samples shown in Figure 3c,d of the main text. The black arrow in panel d) indicates one dinitrene **2**. Scan parameters for all images: V = 100 mV, I = 100 pA and scale bars = 3 nm.

References

[1] H. Iden, F.-G. Fontaine, J.-F. Morin, *Org. Biomol. Chem.* **2014**, *12*, 4117–4123.
[2] B.-C. Liao, B.-H. Jian, M.-J. Wu, J.-T. Lee, *ACS Appl. Energy Mater.* **2023**, *6*, 8581–8589.
[3] F. J. Giessibl, *Review of Scientific Instruments* **2019**, *90*, 011101.
[4] L. Gross, F. Mohn, N. Moll, P. Liljeroth, G. Meyer, *Science* **2009**, *325*, 1110–1114.
[5] I. Horcas, R. Fernández, J. M. Gómez-Rodríguez, J. Colchero, J. Gómez-Herrero, A. M. Baro, *Review of Scientific Instruments* **2007**, *78*, 013705.
[6] J. P. Perdew, K. Burke, M. Ernzerhof, *Phys. Rev. Lett.* **1996**, *77*, 3865–3868.
[7] V. Blum, R. Gehrke, F. Hanke, P. Havu, V. Havu, X. Ren, K. Reuter, M. Scheffler, *Computer Physics Communications* **2009**, *180*, 2175–2196.
[8] A. Tkatchenko, M. Scheffler, *Phys. Rev. Lett.* **2009**, *102*, 073005.
[9] A. D. Becke, *The Journal of Chemical Physics* **1993**, *98*, 5648–5652.
[10] P. J. Stephens, F. J. Devlin, C. F. Chabalowski, M. J. Frisch, *J. Phys. Chem.* **1994**, *98*, 11623–11627.
[11] P. Hapala, R. Temirov, F. S. Tautz, P. Jelínek, *Phys. Rev. Lett.* **2014**, *113*, 226101.
[12] P. Hapala, G. Kichin, C. Wagner, F. S. Tautz, R. Temirov, P. Jelínek, *Phys. Rev. B* **2014**, *90*, 085421.
[13] J. I. Mendieta-Moreno, R. C. Walker, J. P. Lewis, P. Gómez-Puertas, J. Mendieta, J. Ortega, *J. Chem. Theory Comput.* **2014**, *10*, 2185–2193.
[14] J. P. Lewis, P. Jelínek, J. Ortega, A. A. Demkov, D. G. Trabada, B. Haycock, H. Wang, G. Adams, J. K. Tomfohr, E. Abad, H. Wang, D. A. Drabold, *physica status solidi (b)* **2011**, *248*, 1989–2007.
[15] J.-D. Chai, M. Head-Gordon, *Phys. Chem. Chem. Phys.* **2008**, *10*, 6615–6620.
[16] Gaussian 16, Revision C.01, M. J. Frisch, G. W. Trucks, H. B. Schlegel, G. E. Scuseria, M. A. Robb, J. R. Cheeseman, G. Scalmani, V. Barone, G. A. Petersson, H. Nakatsuji, X. Li, M. Caricato, A. V. Marenich, J. Bloino, B. G. Janesko, R. Gomperts, B. Mennucci, H. P. Hratchian, J. V. Ortiz, A. F. Izmaylov, J. L. Sonnenberg, Williams, F. Ding, F. Lipparini, F. Egidi, J. Goings, B. Peng, A. Petrone, T. Henderson, D. Ranasinghe, V. G. Zakrzewski, J. Gao, N. Rega, G. Zheng, W. Liang, M. Hada, M. Ehara, K. Toyota, R. Fukuda, J. Hasegawa, M. Ishida, T. Nakajima, Y. Honda, O. Kitao, H. Nakai, T. Vreven, K. Throssell, J. A. Montgomery Jr., J. E. Peralta, F. Ogliaro, M. J. Bearpark, J. J. Heyd, E. N. Brothers, K. N. Kudin, V. N. Staroverov, T. A. Keith, R. Kobayashi, J. Normand, K. Raghavachari, A. P. Rendell, J. C. Burant, S. S. Iyengar, J. Tomasi, M. Cossi, J. M. Millam, M. Klene, C. Adamo, R. Cammi, J. W. Ochterski, R. L. Martin, K. Morokuma, O. Farkas, J. B. Foresman, D. J. Fox, Gaussian, Inc., Wallingford CT, **2016**.
[17] J. Tomasi, B. Mennucci, R. Cammi, *Chem. Rev.* **2005**, *105*, 2999–3094.
[18] G. Scalmani, M. J. Frisch, *The Journal of Chemical Physics* **2010**, *132*, 114110.
